# Robotic exoskeleton assessment of transient ischemic attack

**DOI:** 10.1371/journal.pone.0188786

**Published:** 2017-12-22

**Authors:** Leif Simmatis, Jonathan Krett, Stephen H. Scott, Albert Y. Jin

**Affiliations:** 1 Centre for Neuroscience Studies, Queen’s University, Kingston, ON, Canada; 2 Department of Medicine, Queen’s University, Kingston, ON, Canada; 3 Dept. of Biomedical and Molecular Sciences, Queen’s University, Kingston, ON, Canada; University of Ottawa, CANADA

## Abstract

We used a robotic exoskeleton to quantify specific patterns of abnormal upper limb motor behaviour in people who have had transient ischemic attack (TIA). A cohort of people with TIA was recruited within two weeks of symptom onset. All individuals completed a robotic-based assessment of 8 behavioural tasks related to upper limb motor and proprioceptive function, as well as cognitive function. Robotic task performance was compared to a large cohort of controls without neurological impairments corrected for the influence of age. Impairment in people with TIA was defined as performance below the 5^th^ percentile of controls. Participants with TIA were also assessed with the National Institutes of Health Stroke Scale (NIHSS) score, Chedoke-McMaster Stroke Assessment (CMSA) of the arm, the Behavioural Inattention Test (BIT), the Purdue pegboard test (PPB), and the Montreal Cognitive Assessment (MoCA). Age-related white matter change (ARWMC), prior infarction and cella-media index (CMI) were assessed from baseline CT scan that was performed within 24 hours of TIA. Acute infarction was assessed from diffusion-weighted imaging in a subset of people with TIA. Twenty-two people with TIA were assessed. Robotic assessment showed impaired upper limb motor function in 7/22 people with TIA patients and upper limb sensory impairment in 4/22 individuals. Cognitive tasks involving robotic assessment of the upper limb were completed in 13 participants, of whom 8 (61.5%) showed significant impairment. Abnormal performance in the CMSA arm inventory was present in 12/22 (54.5%) participants. ARWMC was 11.8 ± 6.4 and CMI was 5.4 ± 1.5. DWI was positive in 0 participants. Quantitative robotic assessment showed that people who have had a TIA display a spectrum of upper limb motor and sensory performance deficits as well as cognitive function deficits despite resolution of symptoms and no evidence of tissue infarction.

## Introduction

The term transient ischemic attack (TIA) suggests that people that have them will acquire no lasting impairments, and indeed this is the original definition of the condition (symptom resolution in <24 hours). However, a significant proportion of people with TIA become disabled by 90 days for reasons that are unclear [1]. One possible reason for this is that people who have experienced TIA—despite being apparently symptom-free—may in fact have subclinical neurological deficits leading to cognitive, sensory and motor dysfunction. Previous work has shown that people with TIA have cognitive impairment which can be lasting [1–4]. However, sensorimotor dysfunction after TIA has not been well described, in part because the deficits may be subtle enough that common bedside clinical exam techniques do not detect them. There is increasing recognition that TIA is not just a risk marker of future stroke, but also a potential marker of functional decline [1]. Currently, we lack the tools to understand the cognitive and motor dysfunction that may contribute to this deterioration. Therefore, there is a need to develop newer comprehensive methods to assess a wider spectrum of neurological deficit in people who have experienced TIA. Here, we use robotic technology to answer the question of whether or not people who have had a TIA have quantifiable sensorimotor impairment after their symptoms are supposed to have resolved. Our goal is to quantify potential upper limb proprioceptive and motor dysfunction, as well as cognitive dysfunction, within 2 weeks of TIA.

We hypothesized that relative to control subjects, a single centre prospective cohort of people with TIA would show motor behavioural deficits detectable up to two weeks after symptom resolution. Secondary to this, we also hypothesize that people who have had a TIA will perform normally on other clinical tests of function, because normal function is the expected outcome of a TIA. We used the KINARM exoskeleton robot (BKIN Technologies Inc., Kingston, Canada), which has been extensively validated for use with stroke patients [5–10]. We assessed aspects of executive function (set switching, response inhibition), spatial working memory as well as upper limb proprioceptive and motor function in tasks that correlate to functional independence [10]. Performance of participants who had TIA was quantified relative to a large database of volunteer control subjects corrected for age. With the addition of bedside clinical assessment of cognitive and motor function we were then able to describe a spectrum of motor and cognitive behaviour impairments in individuals who have had a TIA.

## Methods

### Participants and clinical assessment

Participants were recruited from the Stroke Prevention Clinic at Kingston General Hospital in Kingston, Ontario, Canada. All participants were assessed within 2 weeks of TIA. Inclusion criteria for this study were a diagnosis of TIA, the ability to understand the clinical and robotic assessment tasks, normal vision and no injury limiting movement of the upper extremity. TIA was defined as a transient neurological impairment of suspected vascular origin lasting less than 24 hours with a score of 0 on the National Institutes of Health Stroke Scale (NIHSS) and no neurological deficit on examination by an experienced stroke neurologist. We used the traditional definition of TIA that does not account for evidence of acute tissue infarction because we could not acquire DWI scans for all participants. The Queen’s University Ethics Review Board approved this study, and participants provided written informed consent prior to their participation. All participants with TIA had a baseline non-contrast CT scan of the head within 24 hours of TIA, and a subset had a DWI scan performed within ten days of TIA onset. All participants with TIA had vascular imaging by CT angiography, MR angiography, or carotid Doppler ultrasound. Small vessel ischemic disease on CT scan was assessed using the age-related white matter change (ARWMC) [11] score in all participants with TIA. Brain atrophy was assessed using the cella-media index (CMI) [12] on CT scan. These measurements were done by two of us (JK and AYJ) without knowledge of the clinical assessment or performance in robotic testing. Infarction was assessed using either DWI scan (when available) or CT in all participants with TIA by a stroke neurologist (AYJ) who was blinded to the robotic assessment and clinical scores.

In addition to examination by an experienced stroke neurologist, all participants underwent the following tests: the Behavioural Inattention Test [13] (BIT), Purdue Pegboard Test [14] (PPB), Montreal Cognitive Assessment [15] (MoCA) and the Chedoke-McMaster Stroke assessment (CMSA) for the arm [16,17]. For the latter, individuals with TIA and control volunteers (n = 106) either passed or failed based on their ability to complete stage 7 of the CMSA arm and hand inventories. The order of clinical examinations was consistent for all participants with TIA and controls. Corrected vision was allowed in all examinations.

### Robotic assessment

**Fig 1:**
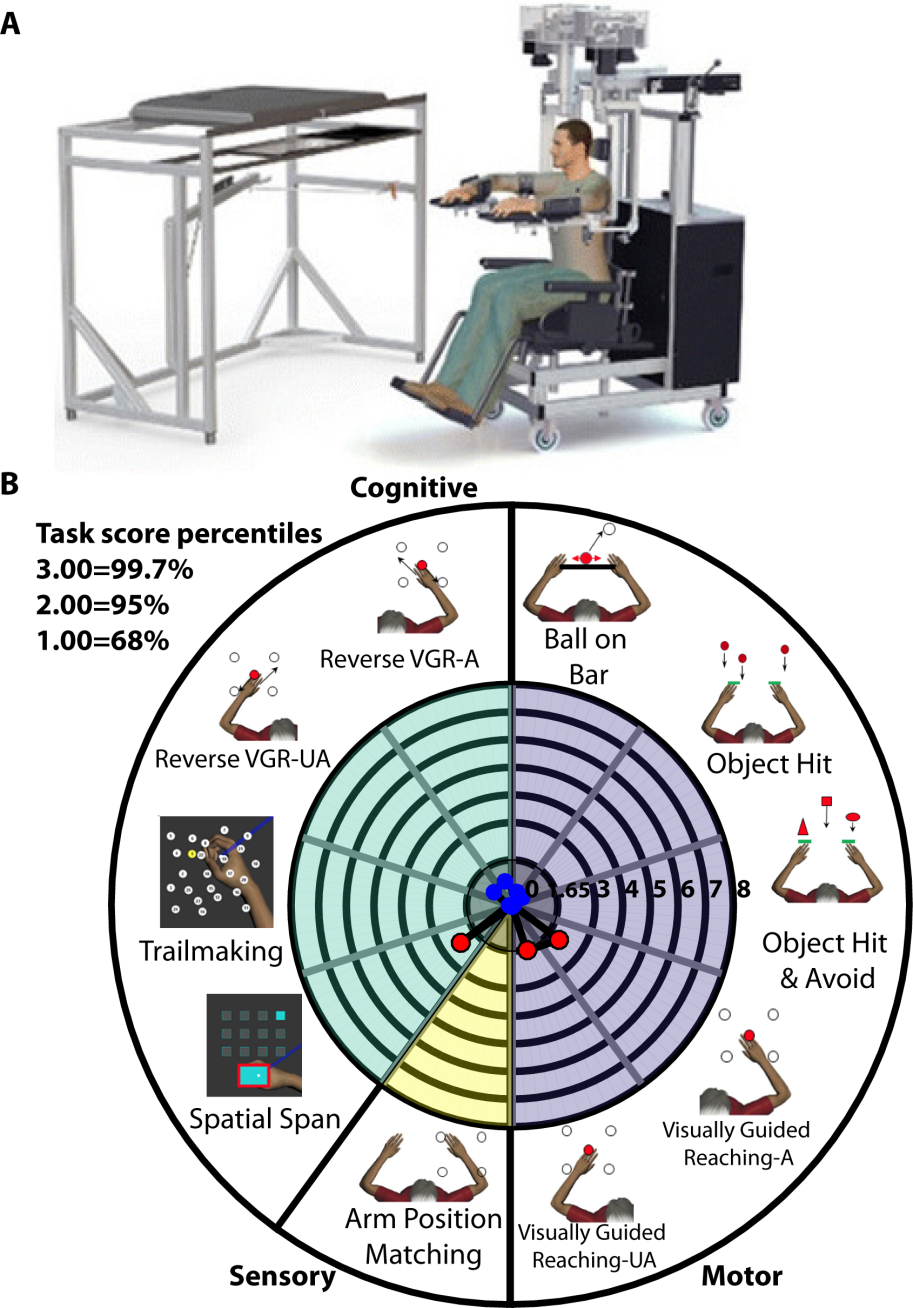
The KINARM robotic exoskeleton is used to assess a variety of upper limb motor and proprioceptive tasks as well as tasks assessing cognitive function. **A.** KINARM robotic exoskeleton. Participants sit in the exoskeleton with their arms supported by troughs. The robot allows horizontal planar movement of the upper limbs. Feedback on tasks is provided via a virtual reality display. Visual feedback of the hands is occluded using a screen. **B.** There are 8 standard tasks, encompassing motor (4 tasks), sensory (1 task), and cognitive (3 tasks) domains. Task scores can be represented in terms of percentiles of performance (1 = 68.3%, 1.96 = 95%).

Quantitative assessment of participants with TIA was performed using the KINARM exoskeleton robot (BKIN Technologies, Kingston, ON). Eight KINARM tasks were used to assess upper limb proprioceptive and motor function, as well as cognitive function. Participants with TIA were seated in the KINARM exoskeleton robot which allows 2D horizontal planar arm movement (Fig 1). A virtual reality system was used to display spatial goals/objects in the horizontal workspace of the arms. A screen obscured visual feedback of the hands, making patients rely on fingertip position feedback which was provided on the display. Tasks were described immediately prior to being performed, and were administered by experienced operators. Task order was determined to ensure that similar tasks were performed consecutively and to minimize fatigue during examination. Controls were part of a database collected by our lab previously. The number of controls per task ranges from 94 to 494 depending on the task (newer tasks have fewer controls). All people included as controls have had no neurological disorder of musculoskeletal injury affecting the upper limb in the last 5 years. The order of tasks was identical for all participants with TIA and controls. Corrected vision was allowed during robotic assessment for subjects and controls.

We used four behavioural tasks to quantify upper limb motor function (Fig 1). Visually-guided reaching (VGR) required participants to reach out and back to spatial targets in an array of either 4 or 8 locations [6]. VGR was changed during the course of data collection from 8 targets to a 4 targets to reduce data collection time. The 8-target variant and the simplified 4-target variant yield similar motor performance data [18]. In the object hit (OH) task, participants tried to hit away virtual balls which fell from the top to the bottom of the video display using paddles virtually attached to their hands [19]. The balls fell from 10 bins spaced evenly across the top of the display and the rate at which balls appeared on the screen increased until the end of the test. The object hit-and-avoid (OHA) task was similar to OH, except participants had to hit two specific target shapes and avoid all other shapes [5]. Ball on bar (BOB) assessed coordination of the two arms. Participants were required to balance a ball on a bar and move the ball to targets in a set amount of time [8].

One task was used to assess proprioceptive function (Fig 1). Arm position matching (APM) task quantified position sense of the upper arm [20]. The robot moved the participant’s affected arm to one of 4 or 9 targets arranged in grid. The participant was asked to mirror the position of the robot-controlled (“active”) arm using their unaffected arm.

The remaining 3 tasks assessed various aspects of cognitive function (Fig 1). Reverse-visually-guided reaching (RVGR) was similar to VGR with the exception that the cursor moved in the direction 180^o^ opposite to the participant’s hand [21–23]. Participants were required to inhibit the automatic response to move the hand towards the target and instead move the hand in the opposite direction to guide the cursor to the target. This task requires several executive processes, including inhibition and attention [24–27]. The trail-making test variant B (TMT-B) tested decision making and task switching [28, 29]. Participants moved their dominant arm along a trail of numbers and letters in sequence (e.g. 1-A-2-B…etc). Spatial span (SS) quantified spatial working memory capacity similar to the Corsi block test [30]. Participants positioned their dominant hand in a demarcated rectangular area on the display screen. This was enforced by a load applied from the robot if the participant’s arm deviated towards the edge of the area. During this time, a series of squares on a 3x4 grid were illuminated in a random sequence. The participant was required to recall the pattern and move their hand to the targets in order. After a successful trial, the next random sequence had an additional target added to the sequence (e.g. after successful recall of three targets, the next trial had four). An unsuccessful trial was followed by a new random sequence with one less target.

### Statistical analysis

Task scores were derived for each behavioural task based on the performance for a large cohort of healthy control. A full description of task score derivation is presented in S1 Methods. Key features of the Task Score is that 0 denotes best performance on the task and increasing values reflect poorer performance. Task Scores follow the same percentiles as ±1SD of a normal distribution (i.e. 1 = 68.3%, 2 = 95.4%, etc.). Task Scores > 1.96 indicated impaired performance on the task.

Other statistical analyses were performed using custom-written Matlab scripts (Mathworks, Natick, MA). T-tests were performed to assess the significance of relationships between clinical variables (CMSA, MoCA, BIT, PPB), imaging measures (CMI, ARWMC) and robotic task performance. One-way ANOVA was performed to determine whether or not people who received DWI scans performed differently on robotic tasks than those who did not receive scans (scan-positive or scan-negative were the groups).

## Results

Summary clinical scores and demographics of individuals with TIA are presented in **Table 1.** Twenty-two participants with TIA were recruited; 21 had TIA with NIHSS = 0 and 1 had TIA but also NIHSS = 4 due to bilateral leg weakness from pre-existing diabetic lumbosacral radiculoplexus neuropathy which does not affect upper limb function. Mean age was 67.1±11.1 years old. Fifty percent of participants were female and 95.5% were right-handed. Symptoms involved the right side of the head and/or body in 54.5% of participants, left in 40.9% of participants, or were non-lateralizing in 4.5% of participants (e.g. truncal ataxia in 1 of 22 patients). Summary clinical data are presented in **Table 2.** Complete clinical data are presented in supplementary **S1 Table.**

**Table 1:** Summary clinical information and demographics of our TIA cohort.

Mean education		12
Comorbidities		
	Hypertension	45.5%
	DM Type 2	4.5%
	Smoking	13.6%
	Prior stroke	0%
	Prior MI	13.6%
	OSA	0%
TIA/Stroke etiology		
	POCS	13.6%
	PACS	77.3%
	LACS	18.2%
	TACS	0%
ARWMC		11.8±6.4
CMI		5.4±1.5
DWI-positive lesion		0%

DM = Diabetes Mellitus; OSA = Obstructive Sleep Apnea; MI = Myocardial Infarction. Stroke abbreviations follow TOAST criteria: POCS = Posterior Circulation Syndrome; PACS = Partial Anterior Circulation Syndrome; LACS = Lacunar Circulation Syndrome; TACS = Total Anterior Circulation Syndrome.

**Table 2:** Individual clinical and demographic information for our cohort of people with TIA.

Sex (% female)	50.0
Dominant hand (% right-handed)	95.5
Affected side (% right-affected)	54.5
CMSA-Left arm (% with normal performance)	36.4
CMSA-Right arm (% with normal performance)	40.9
MoCA (average±SD points)	26.8±4.1
CMI (average±SD)	5.4±1.5

CMSA = Chedoke-McMaster Stroke Assessment; MoCA = Montreal Cognitive Assessment; CMI = Cella-Media Index.

The Purdue pegboard (PPB) and behavioural inattention test (BIT) scores were within the normal range in all participants (means were 22.6±5.7 and 143.3±2.8, respectively). All controls (n = 106) got the maximum score on stage 7 of the CMSA arm inventory (expected score for controls). However, 12/22 (54.5%) people with TIA performed below this level several days after TIA. The mean MoCA score was 26.8±4.1, and 8/22 (36.4%) participants had a score of < 26. The mean age-related white matter change (ARWMC) score was 11.8 ± 6.4 (range 1 to 27). The mean cella-media index (CMI) in all patients was 5.4 ± 1.5 with only one individual having CMI < 4.1 (abnormal). In a subset of 13 people with TIA who underwent DWI scan, none showed a restricted diffusion lesion. Neither the ARWMC score nor CMI correlated to performance on MoCA, BIT, PPB, or pass/fail on CMSA (all p>0.05). Furthermore, there were no correlations between ARWMC and CMI with any robotic assessment tasks. Correlation coefficients between imaging variables and robotic assessment tasks were between 0.55 to -0.45.

**Fig 2:**
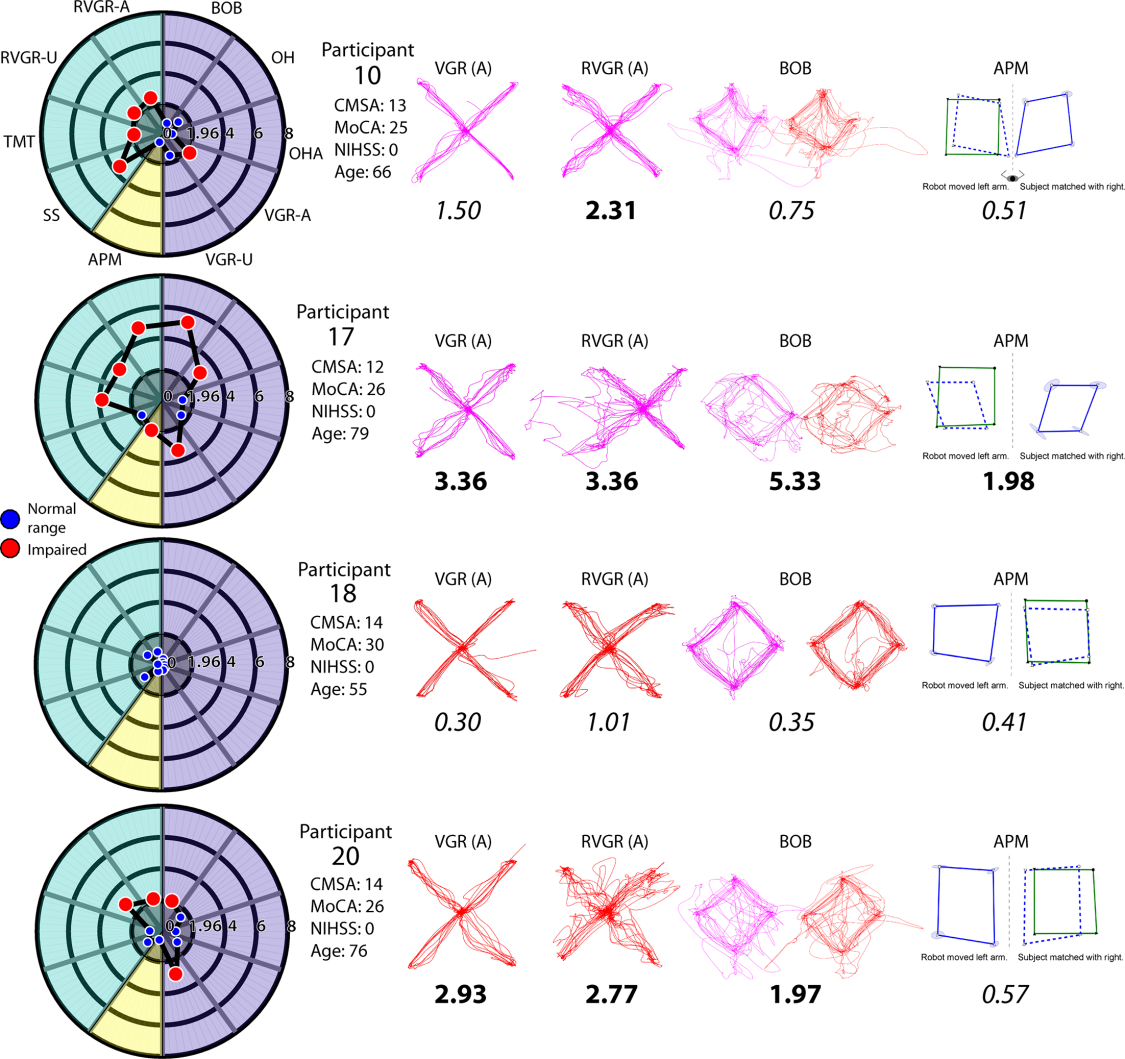
People with TIA showed several individual patterns of impairment after two weeks. Four examples of participants (10, 17, 18 and 20) with different levels of impairment on multiple tasks are shown, to demonstrate the variety observed in the study. Radar plots for each participant are given that summarize task scores for all 8 KINARM tasks. Kinematic data are shown for four tasks (VGR, RVGR, BOB, and APM) and corresponding Task Scores are provided (**below the 5**^**th**^ **percentile** and *within the normal range*).

Robotic assessment of people with TIA demonstrated impairment on tasks testing upper limb motor and proprioceptive performance, as well as cognitive function **(Table 3 and S3 Table)**. We observed a great variety of impairments across the cohort, and we show a small sample of these to demonstrate these individual differences (Fig 2). Seven out of 22 participants with TIA (31.8%) performed below the 5^th^ percentile of controls on at least one motor task. Six participants with TIA (27.3%) were unable to move either arm accurately in a simple reaching task (VGR) using either their affected- or unaffected arm. All participants with TIA were able to use their arms to hit moving targets accurately in Object Hit (OH task). Two participants with TIA (9.1%) were unable to discriminate between targets to be hit and those to be avoided in the Object Hit and Avoid task (OHA). Coordinated use of both arms to move an object to a target in Ball On Bar (BOB) was impaired in 3 participants with TIA (27.3%). This task was completed by 11 participants. Perception of arm position in the Arm Position Matching task (APM) was impaired in 4 people with TIA (18.2%). Cognitive tasks assessing task-switching (Trail Making Test-part B; TMT-B), response inhibition (Reverse Visually Guided Reaching; RVGR), and working memory (Spatial Span; SS) were impaired in 9.1%, 61.5%, and 27.3% of people with TIA, respectively. Thirteen participants completed SS and RVGR. When we compared participants who had received DWI-MRI scans and those who had not, there was no significant difference in robotic task performance (p = 0.73).

**Table 3:** Task score summaries for all participants.

Task	Impairment rate (% below 5^th^ percentile of performance)
VGR-A	13.6
VGR-U	13.6
OH	0
OHA	9.1
BOB	27.3
RVGR-A	46.2
RVGR-U	61.5
TMT-B	9.1
SS	27.3
APM	18.2

Percentages of participants with TIA that performed below the 5^th^ percentile of controls.

Some participants may have been impaired the robotic tasks by random chance given that our threshold for impairments was the 95% level performance of healthy controls and subjects performed 6 or 10 tasks. For example, 26% of healthy controls are predicted to perform poorly on 1 of 6 tasks and 40% are predicted to perform poorly on 1 of 10 tasks. We found that, of the 13 people with TIA that performed all 10 tasks, 9 (69%) performed poorly in a least 1 task. This is significantly higher than expected by random chance (p<0.05, binomial proportion test). Furthermore, only 1 in 8000 healthy subjects should be identified as impaired on 3 tasks, whereas we found 7 (32%) of the subjects failed 3 tasks or more. Thus, these 7 subjects clearly have impairments that are beyond that expected by random chance (p<0.0001).

We analyzed the correlations between performances across all tasks to assess collinearity. Because we wished to identify variables that are redundant, we set a cut-off for the R^2^ value at 0.7. We found R^2^ values of over 0.7 between RVGR-Affected arm and RVGR-Unaffected arm (R^2^ = 0.75); BOB and RVGR-Unaffected arm (R^2^ = 0.70). All other R^2^ were below 0.60 and most of these were below 0.25. Some task pairs such as OH and BOB were almost entirely unrelated (R^2^ = 0.0001). Therefore, 2 task pairs out of a possible 45 unique pairs (no self-correlations or replication of pairs), or 4.4%, had interdependence at the level of R^2^ = 0.7 or higher. A complete R^2^ value summary is presented in supplementary **S4 Table.**

## Discussion

In a single centre cohort of people with transient ischemic attack (TIA), robotic-assisted assessment revealed that many people who have had a TIA display a spectrum of upper limb motor and proprioceptive dysfunction as well as cognitive dysfunction despite resolution of symptoms. The proportion of people in the TIA cohort that performed all robotic tasks at a control-like level was significantly lower than the expected value for controls. In addition, 12/22 (54.5%) participants were unable to perform simple motor tasks involving the upper limb in the Chedoke-McMaster Stroke Assessment (CMSA). This motor dysfunction was not explained by impairment on cognitive screening, age-related white matter change (ARWMC), or cerebral atrophy (CMI). This study shows that in addition to known cognitive impairments, acquisition of disability, and risk of recurrent stroke [1, 2, 31], many people with TIA are impaired in their ability to plan and execute sensorimotor tasks.

Motor impairments such as those we detected with the KINARM and using the CMSA have not been quantified in people with TIA previously. Nearly one-third of participants performed below the 5^th^ percentile of the control group on visually guided reaching. This rate of impairment is far higher than would be expected if TIA symptoms had resolved and there was not a lasting effect. The impairment rate of 31.8% for any motor task further emphasizes that there are considerable motor deficits in this population which currently escape detection upon traditional bedside examination. None of the TIA cohort were impaired in the object hit task, which is counterintuitive given the relatively high rate of impairment in the visually guided reaching task. This may be an effect of our small sample size. It may also be because participants that were impaired in visually guided reaching (VGR) were only impaired in one arm. Therefore, the other, less-impaired, arm may have compensated and allowed them to perform object hit within the normal range. We also observed that 54.5% of participants performed below the level of controls on stage 7 of the CMSA. This rate is higher than for robotic tasks testing motor function only. However, this may reflect that some CMSA tests assess both the arm and hand together (e.g. bounce and catch a ball) whereas our robotic tasks only assess arm function. The coarseness of the CMSA scoring may also relate to the observed failure rate difference compared to KINARM tasks. Robotic assessment provides a continuous and more comprehensive score for performance on a given task. Robot-based tasks are limited to the horizontal plane and weight support of the limb is provided during motor function. It may be beneficial to develop robot-based tasks that require coordinated action of the arm and hand, as well as to add weight support, particularly for these individuals with relatively moderate motor impairments. Accurate detection of motor impairment after TIA in the future will be important due to the association between motor symptoms and an increased risk of subsequent stroke [32].

In contrast to motor impairment, cognitive impairment in TIA is well-described and our observations support those of others. Deficits have been identified in attention, working memory, and processing speed which can persist as long as 90 days [3, 33] after TIA. We found that a similar proportion of people with TIA in our study displayed cognitive impairments as in previous studies [2]. Almost 32 percent were impaired on the Montreal Cognitive Assessment (MoCA), a screening tool that assesses a range of cognitive functions including memory and set-switching. Our robot-based cognitive tasks also identified subjects impaired on specific cognitive functions. Spatial span (SS) and the trail making test part B (TMT-B) were impaired in 27.3 and 9.1%, respectively. Similarities in performance on spatial span, a robot-based memory task, and MoCA is not surprising given the latter is itself heavily weighted towards memory [15].

Of particular interest is the observation that most individuals assessed in the reverse-visually guided reaching task (RVGR) task were identified as impaired. Importantly, the number of participants that performed below the 5^th^ percentile of healthy controls on this task was substantially higher than VGR, which is a very similar task. The level of variability in VGR explained by RVGR was also low, and therefore these two tasks are relatively independent. It is possible that this is because reverse VGR engages multiple regions involved in inhibition and executive control which VGR does not. Reverse reaching requires executive control to inhibit the automatic response to reach towards a spatial goal and instead generate a motor response in the opposite direction in order to move the cursor to the target. Other studies have shown that individuals with mild cognitive impairments are also commonly impaired in this task [21–23]. The wide variety of neural substrates engaged by this task makes it able to identify a possible impairment although unable to identify the exact location of the injury or damage. By including other tasks that test different functions, a more specific picture of impairment may be painted for an individual [34].

Cognitive and motor impairment in people with TIA has been associated with white matter disease, cerebral atrophy and prior infarction. However, imaging features alone cannot yet specifically describe aspects of post-stroke cognition. White matter lesion burden and medial temporal lobe atrophy have been associated with executive and memory dysfunction at one year [35]. Severity of periventricular white matter lesion burden due to small vessel disease has also been associated with poor processing speed and memory, possibly due to interruptions in cortico-subcortical information loops [36]. After accounting for the influences of age, gender and stroke severity, the relationship between white matter changes and cognitive impairment becomes less clear. In a recent study by Mai et al [37], there was no association between ARWMC and MoCA scoring. This may be due to the large variability in ARWMC. Others have noted that after controlling for demographic and vascular risk factors, only temporal lobe atrophy is associated with cognitive impairment [38]. Rasquin et al suggested that only the presence of infarction on CT scan predict cognitive dysfunction after stroke [39]. In our study, neither ARWMC nor CMI correlated to upper limb motor or proprioceptive dysfunction, or cognitive dysfunction. In a subset of participants who underwent DWI scans, none had an acute ischemic lesion and there was no statistical distinction between individuals who had received DWI scanning and those who had not. This suggests that the substrate for motor behavior deficits in people who have had a TIA cannot be explained by imaging features alone.

Disability in people with TIA is a frequent occurrence [1]. Additionally, half of people with TIA experience symptoms of depression and decreased quality of life [40]. Therefore, it will be important to further investigate correlates to changes in quality of life for people who have had a TIA. A first step will be the early detection of motor impairments after TIA, which are associated with a significantly increased risk of subsequent stroke [34].

It is important to acknowledge the limitations of this study. The first is that we do not have DWI results for all patients. This makes it difficult to conclude that all patients did not have infarcts present which would have affected their performance. However, task scores for the 13 individuals with a DWI scan were statistically indistinguishable from those who had not been scanned. Two further limitations are that not all patients completed all tasks, and that we had a small sample size. The primary goal of our work was to determine whether or not people who have had a TIA have quantifiable impairments on our robotic assessment tasks. We were able to identify impairments in people that have had a TIA, although we could not capture the full spectrum of impairments with our small sample. This will be addressed in future works, which will allow us to perform more detailed analyses of impairment patterns in this population. Finally, we must acknowledge that the condition of people with TIA may change within the 2 week window between TIA and assessment. We accept this limitation, as our study was not intended to differentiate changes within the 2-week timeframe. Instead, we asked the question of whether or not there would be quantifiable sensorimotor and cognitive impairments at up to 2 weeks. We were able to identify impairments after this window, which runs contrary to the traditional definition of TIA which requires symptom resolution within 24 hours.

There is a pressing need to discern specific patterns of impairment in people who have had a TIA to identify individuals at an elevated risk of future cerebral ischemic events. Here we have demonstrated two main findings. The first is that people who have had a TIA display quantifiable impairments in sensorimotor and cognitive domains (executive function, spatial working memory, and set-switching). The second is that these impairments are present beyond the 24 hour period normally ascribed to TIA symptom resolution. Current clinical assessments are subjective, and there is a need for quantitative assessment to provide additional information which will reduce error. Impairments detected on MoCA and CMSA in a population of people with TIA have been quantified using the KINARM robot. Comprehensive robotic assessment was able to provide additional details regarding possible mechanisms of impairment and the degree of impairment as compared to healthy individuals. Quantitative robotic assessment may prove to be useful in the future for providing specific information to improve therapeutic decision-making, with the ultimate goal of improving patient care. Future work will be concerned with identifying the relationship between robotic task performance and real-world correlates of disability such as the Barthel Index or the modified Rankin Scale. This will allow us to discuss our findings in a translational and clinically-relevant context.

## Supporting information

S1 Methods (DOC)

S1 Fig The distribution of task scores approximates a Normal cumulative distribution function (CDF) except that the task score is exclusively positive. Task scores have comparable percentile representations to the standard Normal distribution CDF. Task scores of 1, 2, and 3 represent percentiles of 68.3%, 95.4%, and 99.7%.
(DOCX)

S1 Table Complete demographic characteristics for all people with TIA in our cohort. (DOCX)

S2 Table Correlation coefficients between robotic assessment tasks and imaging variables. (DOCX)

S3 Table Complete task score summary for all participants in our cohort of people with TIA. (DOCX)

S4 Table Complete R^2^ value summary for inter-task explanation. (DOCX)
