# Supplementary material for: Robotic exoskeleton assessment of transient ischemic attack
Source: PLoS One. 2017 Dec 22;12(12):e0188786. doi: 10.1371/journal.pone.0188786 (PMC5741219; doi:10.1371/journal.pone.0188786)
Supplement: S1 Methods — (DOC) [file pone.0188786.s001.doc]

**Supplement Methods**

The KINARM exoskeleton robot captures a multitude of parameters in each task, creating more than a 100 metrics of performance across the tasks (See KINARM Standard Tests-Parameter Tables, BKIN Technologies). Here, we compute a Task Score by condensing robotic task data down to a single score. This method compares the performance of people in our TIA cohort to performance from a database of healthy controls. The Task Score provides a global measure of performance for a given participant on a task and was designed such that 0 denotes best performance and increasing values represents poorer performance. Healthy controls used to construct this Task Score completed up to 8 tasks. The control database for each task varied in size from 94 to 494, as newer tasks have been tested in fewer control participants.

The first stage to develop the Task Score converted task parameters into standardized Z-scores. To begin, task parameters for healthy control subjects were converted to Z-scores using Box-Cox transforms [1]. Linear regressions were then used to consider the effects of age on task performance. Distributions were then tested for normality and Box-Cox equations were adjusted if necessary to attain normality. Z-scores ≤ -3.29 or ≥ +3.29 were deemed to be outliers and removed from the dataset, as they can have a substantial impact on the Box-Cox transforms (on average ~1% of subjects were removed per parameter). Outliers were removed because our control sample was random and community-based. Therefore, it was possible that some individuals in our healthy cohort had undiagnosed underlying conditions that negatively affected performance. The process of de-skewing and outlier removal was repeated until there were no remaining outliers and the transformed data (including regressions for age) were normally distributed.

The second stage required these task parameter Z-scores to be transformed such that best performance on a metric equaled 0 and increasing values reflected poorer performance. Best performance for a given metric could reflect large negative Z-scores (e.g. reaction time for visually guided reaching), large positive Z-scores (e.g. objects hit for the object hit task) or values near zero (e.g. parameters that compare limb symmetry, such as hand speed difference for object hit). Parameters where best performance was a large negative Z-score were shifted using **equation 1**, in which the error function (*erf*) and its inverse (*erf^-1^*) are defined in a manner similar to that described by Winitzki [2] (these transformed Z-scores are referred to as Zeta-scores). Parameters where best performance was a large positive Z-score had their sign reversed before using equation 1. Zeta-scores for parameters where best performance was near 0 simply equalled Z-scores values.

The final stage of processing calculates Task Scores based on performance of healthy controls. To begin, the root-mean square (RMS) of the Zeta-scores for healthy controls were calculated (equation 2), and then transformed into a normal distribution using Box-Cox Equations (and tested for normality). These RMS Z-scores were then transformed to a Task Score using equation 1, where 0 equals best performance and increasing values reflect poorer performance. Outliers were again removed if the Task Score for healthy subjects was ≥ 3.29 and the above process was repeated until no further outliers were removed (on average, ~1% of subjects removed). The Task Score distribution for healthy controls shares key percentiles with the Normal distribution: task scores of 1, 2, and 3 are the 68.3^rd^, 95.4^th^, and 99.7^th^ percentiles for healthy subject performance. These Task Scores were provided as part of the data analysis routines in KINARM Standard Tests™ (Version 3.6, BKIN Technologies). Performance metrics for people with TIA were computed using these analysis routines and participants were identified as impaired for Task Scores were ≥ 1.96, representing the 95% percentile performance for healthy controls.


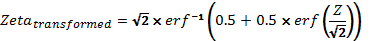
 **eq. 1**


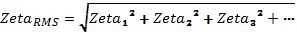
 **eq. 2**

1. Box GEP and Cox DR. An analysis of transformations. J R Stat Soc B Methodol. 1964; 26(2): 211-252.

2. Winitzki S. A handy approximation for the error function and its inverse. 2008 February. Accessed on 4 Oct 2017 from https://www.academia.edu/9730974/A_handy_approximation_for_the_error_function_and_its_inverse.
