# Supplementary material for: Robotic exoskeleton assessment of transient ischemic attack
Source: PLoS One. 2017 Dec 22;12(12):e0188786. doi: 10.1371/journal.pone.0188786 (PMC5741219; doi:10.1371/journal.pone.0188786)
Supplement: S1 Fig — Task scores have comparable percentile representations to the standard Normal distribution CDF. Task scores of 1, 2, and 3 represent percentiles of 68.3%, 95.4%, and 99.7%. (DOCX) [file pone.0188786.s002.docx]

**
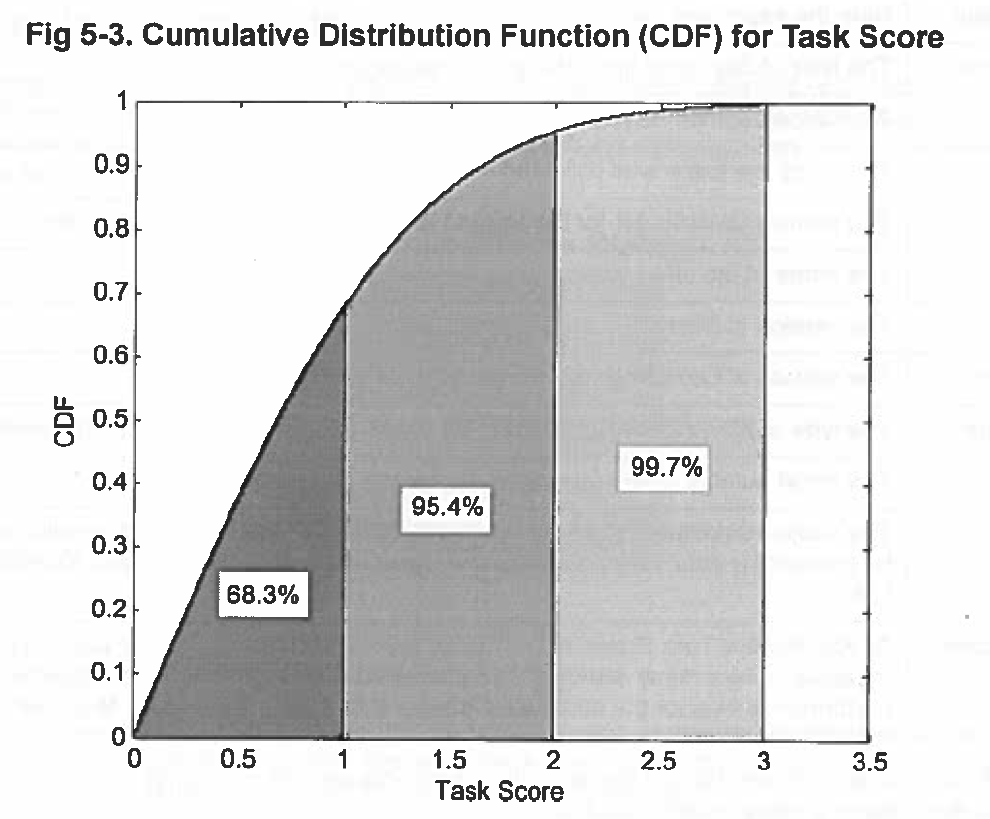
**

**S1 Fig.** The distribution of task scores approximates a Normal cumulative distribution function (CDF) except that the task score is exclusively positive. Task scores have comparable percentile representations to the standard Normal distribution CDF. Task scores of 1, 2, and 3 represent percentiles of 68.3%, 95.4%, and 99.7%.
