# Supplementary material for: Robotic exoskeleton assessment of transient ischemic attack
Source: PLoS One. 2017 Dec 22;12(12):e0188786. doi: 10.1371/journal.pone.0188786 (PMC5741219; doi:10.1371/journal.pone.0188786)
Supplement: S1 Table — (DOCX) [file pone.0188786.s003.docx]

**S1 Table.** Complete demographic characteristics for all people with TIA in our cohort.

| Participant number | Gender | Dominant hand | Affected side | Age | DWI | NIHSS | CMSA-L Arm | CMSA-R Arm | MoCA |
| --- | --- | --- | --- | --- | --- | --- | --- | --- | --- |
| 1 | F | R | R | 69 | - | 0 | 7 | 6 | 30 |
| 2 | M | R | R | 55 | - | 0 | 7 | 7 | 30 |
| 3 | F | R | L | 49 | - | 0 | 5 | 7 | 30 |
| 4 | M | R | L | 54 | - | 0 | 7 | 7 | 30 |
| 5 | M | L | R | 66 | - | 4 | 5 | 6 | 22 |
| 6 | F | R | R | 65 | - | 0 | 7 | 6 | 30 |
| 7 | M | R | R | 67 | - | 0 | 7 | 6 | 27 |
| 8 | M | R | L | 75 | - | 0 | 6 | 7 | 20 |
| 9 | F | R | L | 50 | - | 0 | 7 | 7 | 29 |
| 10 | M | R | L | 66 | - | 0 | 7 | 6 | 25 |
| 11 | M | R | R | 85 | - | 0 | 6 | 6 | 16 |
| 12 | M | R | B | 67 | - | 0 | 7 | 7 | 28 |
| 13 | F | R | L | 72 | - | 0 | 6 | 6 | 30 |
| 14 | F | R | R | 60 | -* | 0 | 7 | 7 | 30 |
| 15 | F | R | R | 78 | -* | 0 | 6 | 6 | 30 |
| 16 | F | R | L | 62 | -* | 0 | 7 | 7 | 30 |
| 17 | F | R | L | 58 | -* | 0 | 6 | 6 | 26 |
| 18 | M | R | R | 55 | -* | 0 | 7 | 7 | 30 |
| 19 | F | R | R | 84 | -* | 0 | 6 | 7 | 25 |
| 20 | M | R | R | 79 | -* | 0 | 7 | 7 | 26 |
| 21 | F | R | R | 84 | -* | 0 | 7 | 7 | 25 |
| 22 | M | R | L | 76 | -* | 0 | 7 | 7 | 20 |
| Mean ±SD | 50% female | 95.5% right-handed | 54.5% right-affected | 67.1±11.1 | - | - | 8 | 9 | 26.8±4.1 |

DWI=Diffusion-Weighted Imaging. (-) indicates that DWI scanning was negative for acute ischemic lesions; * indicates that DWI scanning was not performed. NIHSS=National Institutes of Health Stroke Scale. CMSA=Chedoke-McMaster Stroke Assessment; L=left and R=right. MoCA=Montreal Cognitive Assessment.
