# Supplementary material for: Robotic exoskeleton assessment of transient ischemic attack
Source: PLoS One. 2017 Dec 22;12(12):e0188786. doi: 10.1371/journal.pone.0188786 (PMC5741219; doi:10.1371/journal.pone.0188786)
Supplement: S2 Table — (DOCX) [file pone.0188786.s004.docx]

**S2 Table.** Correlation coefficients between robotic assessment tasks and imaging variables.

|  | ARWMC | CMI |
| --- | --- | --- |
| VGR-A | 0.06 | -0.06 |
| VGR-U | 0.33 | -0.19 |
| OHA | 0.23 | -0.03 |
| OH | 0.05 | -0.04 |
| BOB | 0.14 | 0.10 |
| RVGR-A | 0.55 | 0.05 |
| RVGR-U | 0.27 | 0.06 |
| TMT | 0.09 | 0.11 |
| SPS | 0.35 | -0.31 |
| APM | 0.28 | -0.45 |
