# Supplementary material for: Robotic exoskeleton assessment of transient ischemic attack
Source: PLoS One. 2017 Dec 22;12(12):e0188786. doi: 10.1371/journal.pone.0188786 (PMC5741219; doi:10.1371/journal.pone.0188786)
Supplement: S3 Table — (DOCX) [file pone.0188786.s005.docx]

**S3 Table.** Complete task score summary for all participants in our cohort of people with TIA.

| Participant number | VGR-A | VGR-U | OH | OHA | BOB | RVGR-A | RVGR-U | TMT-B | SS | APM | Tasks impaired per person |
| --- | --- | --- | --- | --- | --- | --- | --- | --- | --- | --- | --- |
| 1 | *0.03* | *0.56* | *1.88* | *0.29* | **-** | **-** | **-** | *1.03* | **-** | *1.71* | 0/6 |
| 2 | *0.17* | *0.59* | *0.49* | *0.39* | **-** | **-** | **-** | *0.35* | **-** | *0.65* | 0/6 |
| 3 | *0.30* | *0.76* | *0.96* | *1.13* | **-** | **-** | **-** | *0.78* | **-** | *0.52* | 0/6 |
| 4 | *0.20* | *0.18* | *0.46* | *0.09* | **-** | **-** | **-** | *0.09* | **-** | *0.34* | 0/6 |
| 5 | *1.50* | **2.95** | *1.51* | *0.39* | **-** | **-** | **-** | *1.25* | **-** | *1.60* | 1/6 |
| 6 | *0.70* | *0.51* | *0.98* | *1.52* | **-** | **-** | **-** | *0.88* | **-** | *1.50* | 0/6 |
| 7 | *1.06* | *1.17* | *1.21* | *1.30* | **-** | **-** | **-** | *0.92* | **-** | **2.35** | 1/6 |
| 8 | *0.94* | *1.84* | *1.22* | *0.47* | **-** | **-** | **-** | *1.32* | **-** | *0.65* | 0/6 |
| 9 | **1.99** | *1.86* | *0.27* | *0.21* | **-** | **-** | **-** | *1.37* | **-** | *1.10* | 1/6 |
| 10 | *1.50* | **2.15** | *0.61* | *1.23* | *0.75* | **2.31** | **2.51** | **1.97** | **3.41** | *0.51* | 5/10 |
| 11 | *1.86* | **2.63** | *1.28* | **2.51** | *0.97* | *1.67* | **3.23** | *1.44* | **7.85** | **2.32** | 5/10 |
| 12 | *0.49* | *0.68* | *1.21* | *0.38* | *0.17* | *0.72* | *0.10* | *0.55* | *1.31* | *0.95* | 0/10 |
| 13 | *1.18* | *1.56* | *0.96* | *1.19* | **3.83** | **4.04** | **5.08** | *1.54* | **4.21** | *1.57* | 4/10 |
| 14 | *1.03* | *0.56* | *1.59* | *1.08* | **-** | *1.35* | *1.78* | *0.52* | *0.75* | *0.79* | 0/10 |
| 15 | *0.70* | *0.27* | *1.08* | *1.23* | *0.87* | **3.96** | **2.54** | *0.44* | **2.29** | *0.22* | 3/10 |
| 16 | *0.63* | *0.49* | *1.52* | *0.40* | *0.63* | *0.26* | **2.47** | *1.50* | *0.01* | *0.89* | 1/10 |
| 17 | **3.36** | *1.55* | *1.34* | **2.96** | **5.33** | **3.36** | **4.93** | **3.85** | *1.55* | **1.98** | 7/10 |
| 18 | *0.30* | *0.07* | *0.20* | *0.14* | *0.35* | *1.01* | *0.87* | *0.06* | *1.32* | *0.41* | 0/10 |
| 19 | *0.42* | *0.71* | *0.14* | *0.55* | *1.32* | *1.52* | *1.53* | *0.98* | *1.08* | *1.78* | 0/10 |
| 20 | **2.93** | *1.21* | *0.80* | *1.53* | **1.97** | **2.77** | **2.11** | *0.73* | *1.10* | *0.57* | 4/10 |
| 21 | *1.12* | *1.03* | *1.63* | *0.91* | **-** | *1.56* | *1.23* | *0.99* | **3.15** | *0.11* | 1/10 |
| 22 | *0.56* | *1.39* | *0.71* | *1.11* | *1.10* | **2.21** | **3.58** | *1.83* | **3.33** | **2.00** | 4/10 |
| Task impairment rate (%) | 13.6 | 13.6 | 0 | 9.1 | 27.3 | 46.2 | 61.5 | 9.1 | 27.3 | 18.2 | 17.3 |

(-) indicates that a task was not completed. Tasks score are indicated as either **below the 5^th^ percentile** or *within the normal range*.
