# Supplementary material for: Robotic exoskeleton assessment of transient ischemic attack
Source: PLoS One. 2017 Dec 22;12(12):e0188786. doi: 10.1371/journal.pone.0188786 (PMC5741219; doi:10.1371/journal.pone.0188786)
Supplement: S4 Table — (DOCX) [file pone.0188786.s006.docx]

**S4 Table.** Complete R^2^ value summary for inter-task explanation.

|  | VGRA | VGRU | OH | OHA | BOB | RVGRA | RVGRU | TMT | SPS | APM |
| --- | --- | --- | --- | --- | --- | --- | --- | --- | --- | --- |
| VGRA | 1 |  |  |  |  |  |  |  |  |  |
| VGRU | **0.34** | 1 |  |  |  |  |  |  |  |  |
| OH | 0.01 | 0.02 | 1 |  |  |  |  |  |  |  |
| OHA | **0.45** | 0.12 | 0.06 | 1 |  |  |  |  |  |  |
| BOB | **0.38** | 0.04 | 1.00E-04 | **0.41** | 1 |  |  |  |  |  |
| RVGRA | 0.21 | 0.01 | 0.001 | **0.32** | **0.57** | 1 |  |  |  |  |
| RVGRU | 0.23 | 0.05 | 0.005 | **0.41** | **0.7** | **0.75** | 1 |  |  |  |
| TMT | **0.42** | **0.3** | 0.05 | **0.38** | **0.5** | 0.15 | **0.4** | 1 |  |  |
| SPS | 0.07 | 0.18 | 0.002 | **0.3** | 0.12 | **0.37** | **0.44** | 0.07 | 1 |  |
| APM | 0.05 | 0.18 | 0.03 | 0.18 | 0.11 | 0.0003 | 0.07 | 0.24 | 0.06 | 1 |

R^2^ values over 0.24 are indicated with **bold text** except for values lying on the unity line from top-left to bottom-right. Values are indicated as **0.59≥x≥0.25** and **x≥0.60**.
